# Supplementary material for: Staphylococcus saccharolyticus Associated with Prosthetic Joint Infections: Clinical Features and Genomic Characteristics
Source: Pathogens. 2021 Mar 26;10(4):397. doi: 10.3390/pathogens10040397 (PMC8066136; doi:10.3390/pathogens10040397)

**Supplement Figure S1:** SNP analysis of clade 1 and clade 2 strains. **A**. Based on SNPs in the core genome the phylogeny is shown for strains for clade 1; 13T028 was used as reference strain. **B**. The SNPs and the derived phylogeny of strains of clade 2 is shown; DVP5-16-4677 was used as reference. Pink lines correspond to SNPs, grey lines/regions represent non-core genome regions.


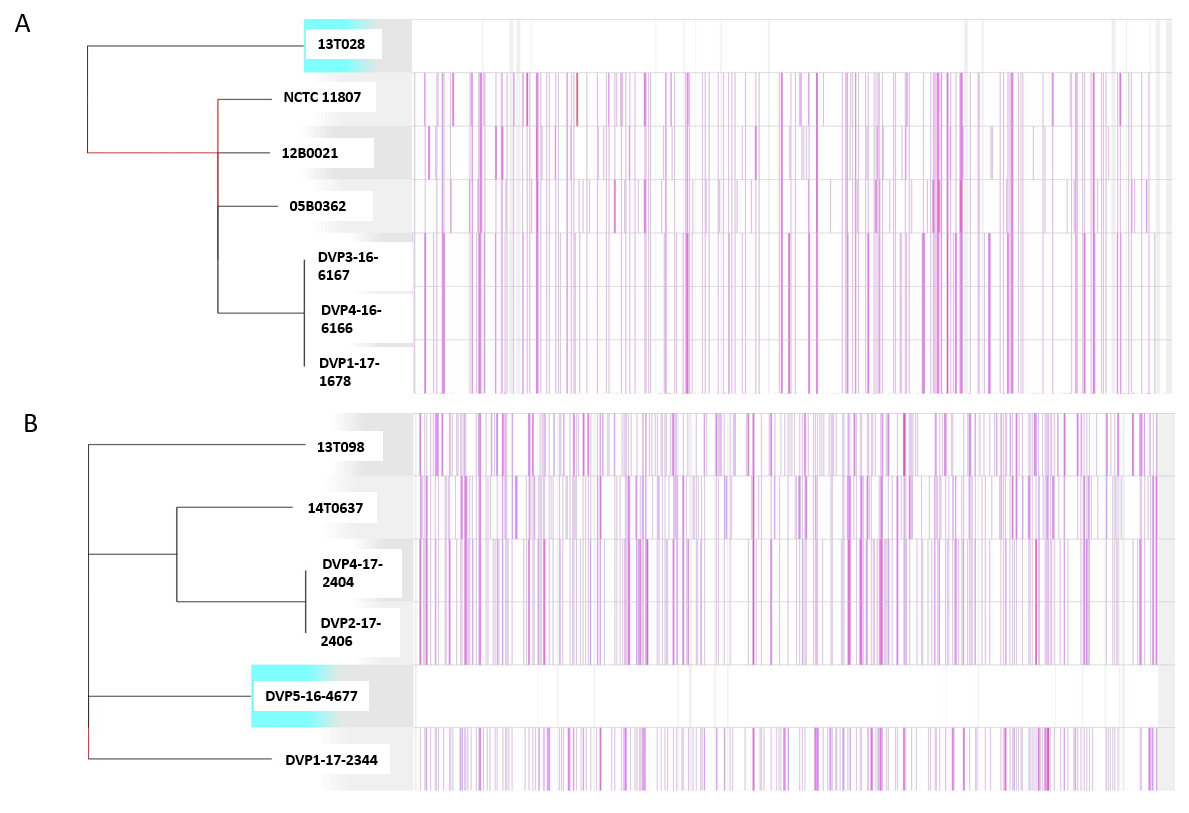

Supplement: Supplementary file 1 [file pathogens-10-00397-s001.zip › figure S1.docx]
